# The Relationship Between Perceived Stress and Social Anxiety in Junior High School Students: The Chain Mediating Role of Psychological Inflexibility and Problematic Social Media Use

**DOI:** 10.3390/bs16060900

**Published:** 2026-06-02

**Authors:** Mengyuan Fang, Zihao Wang

**Affiliations:** 1 Faculty of Education, Huaibei Normal University, Huaibei 235000, China; fangmengyuan0715@163.com; 2 Anhui Engineering Research Center for Intelligent Computing and Application on Cognitive Behavior (ICACB), Huaibei 235000, China

**Keywords:** perceived stress, social anxiety, psychological inflexibility, problematic social media use, junior high school students

## Abstract

Junior high school students represent a high-risk population for social anxiety, and reducing their levels of social anxiety is particularly crucial for their growth and psychological well-being. This study aims to examine the impact of perceived stress on social anxiety among junior high school students and its mediating mechanisms. The research involved 682 junior high school students (345 females; *Mage* = 14.46, *SDage* = 0.50), using the Perceived Stress Scale, Adolescent Social Anxiety Scale, Adolescent Avoidance and Convergence Questionnaire, and Problematic Social Media Use Assessment Questionnaire. The results indicate that perceived stress not only directly and positively predicts social anxiety in junior high school students but also may indirectly be associated with social anxiety through the mediating effect of psychological inflexibility. Further mediation analysis revealed that psychological inflexibility and problematic social media use (PSMU) exerted a chain mediating effect between perceived stress and social anxiety. Specifically, perceived stress triggered psychological inflexibility, which in turn promoted PSMU, thereby ultimately may exacerbate social anxiety among junior high students. This study offers a novel theoretical perspective on understanding the formation of social anxiety in junior high students and provides empirical evidence for schools and families to implement targeted mental health interventions.

## 1. Introduction

Junior high school is a critical period for individuals’ social development. Junior high school students face the dual challenges of peer interaction and social cognitive development (Flanagan et al., 2008). The mismatch between accelerated physical maturation and relatively slow psychological development (B. Z. Chen et al., 2023) makes social anxiety an increasingly prominent mental health issue. Social anxiety (SA) refers to persistent anxiety or avoidance behaviors triggered by others’ evaluation or scrutiny in social situations (Morrison & Heimberg, 2013), and it typically emerges during childhood or adolescence (Y. Zhang et al., 2019). Research indicates that the prevalence of social anxiety in children and adolescents is approximately 20.2% (Ramdhonee-Dowlot et al., 2025). The global prevalence of social anxiety disorder increases with age: 4.7% in children, 8.3% in adolescents, and 17% in youth (Salari et al., 2024). Moreover, junior high school students exhibit significantly higher rates compared to other age groups (Aderka et al., 2012). Recent studies have further demonstrated that perceived stress plays a significant role in adolescent social anxiety (Shao et al., 2025).

Social anxiety has a wide range of negative effects on children’s and adolescents’ social adaptation, such as increasing loneliness (Maes et al., 2019) and impairing the quality of peer relationships (Chiu et al., 2021). Junior high school students with social anxiety possess a negative cognitive processing pattern that distorts their information processing, leads them to avoid social learning opportunities, and impairs their interpersonal skills, ultimately hindering the development of their social adaptation (Davidson et al., 2011).

Although previous studies have revealed the significant impact of social anxiety on the physical and mental development of junior high school students, the internal psychological mechanisms of social anxiety in this population still require further investigation, especially in the context of the increasingly prevalent digital social environment. Research has shown that social anxiety is influenced by a variety of antecedent variables, including internal factors such as negative self-beliefs and fear of negative evaluation (S. Peng et al., 2019; Geng et al., 2025), as well as external factors such as negative life events, parental psychological control, and social support (Bilgin, 2020; Han, 2025; Zhou et al., 2025). Among them, perceived stress has been confirmed by multiple studies as an important risk factor for social anxiety, and there is a significant positive correlation between the two (Blöte et al., 2022; Ni et al., 2016).

Perceived stress refers to an individual’s cognitive appraisal of stressors and can directly affect junior high school students’ executive function (L. Zhang et al., 2025). High levels of stress may not only hinder academic achievement but also impair mental health (Acharya & Sahani, 2022), and these factors further complicate this psychological process (B. Chen et al., 2023). Therefore, to reduce social anxiety and improve the mental health of junior high school students, the present study aims to investigate the association between perceived stress and social anxiety in this population, as well as its underlying psychological mechanisms.

### 1.1. Perceived Stress and Social Anxiety

Perceived stress (PS) is defined as the psychological response generated through cognitive appraisal processes when individuals encounter threatening environmental stimuli (Lazarus & Folkman, 1984). The intensity of perceived stress varies across individuals and has differential impacts on mental health (Pang et al., 2021). Junior high school students undergo multiple transitions in learning styles, interpersonal relationships, and lifestyle patterns, while also coping with pressures from family expectations and social comparison, making them a susceptible population. Previous research has shown a significant positive correlation between perceived stress and social anxiety: the higher the level of perceived stress, the more intense the tension, unease, and fear individuals experience in social situations (von Dawans et al., 2018; Saxena et al., 2021). According to Lazarus and Folkman’s (1987) cognitive appraisal theory of stress, when perceived stress exceeds an individual’s coping capacity, feelings of loss of control and threat perception emerge, which in turn trigger maladaptive symptoms such as cognitive biases and emotional dysregulation (Q. Chen et al., 2025). Therefore, junior high school students are more likely to interpret daily social situations as potential threats, which may trigger excessive fear of negative evaluation and ultimately exacerbate social anxiety on prone to high levels of perceived stress.

### 1.2. The Mediating Role of Psychological Inflexibility

According to the conservation of resources theory (Halbesleben et al., 2014), individuals strive to protect, acquire, and maintain psychological resources. Perceived stress is a risk factor that leads to resource depletion. To alleviate the negative impact of stress, junior high school students must mobilize coping resources. When junior high school students experience high levels of perceived stress, their psychological resources become increasingly depleted. Excessive depletion may then trigger psychological inflexibility (PI), which is a maladaptive coping state characterized by an inability to remain present and flexibly adjust behavior in the face of negative experiences (Kashdan & Rottenberg, 2010).

Research shows that perceived stress significantly and positively predicts psychological inflexibility (Hu & Chen, 2020). Individuals with high psychological inflexibility are more likely to experience anxiety and avoidance in social situations. Junior high school students with psychological inflexibility often hold onto negative thoughts. This happens because they are influenced by negative environments or unhealthy relationships. As a result, they tend to avoid social interactions. This avoidance then reduces their sense of well-being in interpersonal relationships.

### 1.3. The Mediating Role of Problematic Social Media Use

Problematic social media use (PSMU) refers to high-intensity, prolonged engagement with social media that leads to psychological and physiological discomfort (Brand et al., 2016; Savci et al., 2022). For junior high school students, online social media usage has gradually become an equally important relaxation method as real-life social interactions (Li, 2024). The compensatory Internet use model (Kardefelt-Winther, 2014) suggests that individuals may rely on online activities to escape real-life challenges, and that long-term reliance increases the risk of problematic use. Cross-lagged studies have found that problematic online use positively predicts social anxiety (Wang et al., 2017), and excessive mobile social network use is directly correlated with interpersonal anxiety among adolescents (Qin et al., 2018).

### 1.4. The Chain Mediating Role of Psychological Inflexibility and Problematic Social Media Use

There may be an association between psychological inflexibility and problematic social media use. Individuals with psychological inflexibility are prone to maladaptive cognitions—believing they have little value in real life and can only gain respect or success through online platforms (Huang & Chen, 2019). Psychological energy depletion caused by stress may lead junior high school students to reduce high-effort real-world social interactions and shift toward low-effort online interactions. Social media thus becomes a primary avenue for escaping real-life challenges and isolating negative emotions. Empirical evidence shows a significant positive correlation between psychological inflexibility and smartphone addiction among college students (Chou et al., 2017). It is important to note that the effect of PSMU on social anxiety may depend on underlying cognitive factors; simply using social media excessively may not directly exacerbate anxiety unless accompanied by psychological inflexibility.

### 1.5. Current Study

While previous research has separately examined the roles of psychological inflexibility and problematic social media use in relation to stress and social anxiety, no study has tested whether these two factors operate sequentially as chain mediators. More importantly, we propose that PSMU may not function as an independent mediator on its own; rather, it may only exacerbate social anxiety when it is driven by underlying psychological inflexibility. That is, perceived stress first depletes coping resources and induces rigid, avoidant coping patterns (psychological inflexibility), which then pushes adolescents toward maladaptive social media use as an escape. This chain mechanism has not been previously tested, and its confirmation would offer a more nuanced understanding of how environmental stress translates into social anxiety in the digital age.

**Figure 1:**
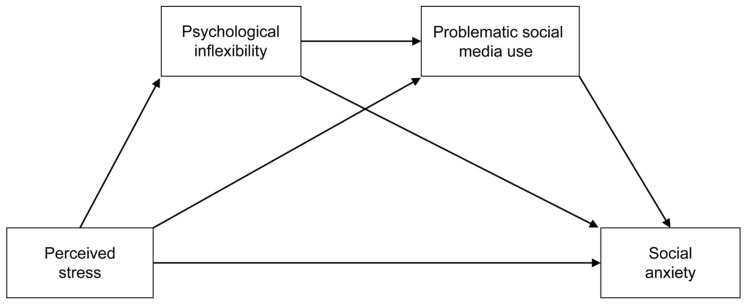
Hypothesis Model.

Therefore, this study proposes the following hypotheses based on the cognitive appraisal theory of stress, the conservation of resources theory, and the compensatory internet use model. A visual model is presented in Figure 1.

**Hypothesis 1.** 
*Perceived stress is positively associated with social anxiety in junior high school students.*

**Hypothesis 2.** 
*Psychological inflexibility mediates the relationship between perceived stress and social anxiety in junior high school students.*

**Hypothesis 3.** 
*Problematic social media use mediates the relationship between perceived stress and social anxiety in junior high school students.*

**Hypothesis 4.** 
*Psychological inflexibility and problematic social media use act as chain mediators between perceived stress and social anxiety in junior high school students.*

## 2. Materials and Methods

### 2.1. Participants

This study used a cluster random sampling method with classes as the sampling unit to select 750 participants from junior high schools in Anhui Province, China. Considering that ninth-grade students need to endure the tremendous pressure brought by the high school entrance examination (Zhongkao), we excluded them from this study and only targeted seventh- and eighth-grade students, so as to avoid extra burden and potential confounding effects of exam-related stress. After excluding invalid questionnaires, 682 valid responses were obtained, resulting in a 90.93% effective response rate. Participants were categorized by geographic distribution: 545 (79.9%) were from urban areas, and 137 (20.1%) were from rural regions. Gender distribution included 337 males (49.40%) and 345 females (50.60%). Grade distribution included 368 students (53.96%) in Grade 7 and 314 students (46.04%) in Grade 8. Among these participants, 276 (40.5%) were only children and 406 (59.5%) had siblings. Mean age was 14.46 ± 0.50 years.

### 2.2. Procedure

Before the survey, all surveyed participants completed a consent form and were informed that participation was completely voluntary and that they had the right to withdraw from or suspend the process anytime. With the consent of both class teachers and students, the researcher conducted a standardized paper-and-pencil questionnaire survey on a class-by-class basis.

After the survey, questionnaires were collected, organized, and screened for invalid responses (e.g., missing responses >20%, obvious repetitive patterns, or contradictory answers), with valid data entered into the system after a final review. All the above procedures were performed in accordance with the ethical standards of scientific research and were approved in advance by the Ethics Approval Committee of Huaibei Normal University (issue: HBNU-202612).

### 2.3. Variables and Measures

#### 2.3.1. Perceived Stress Scale

Perceived Stress was assessed using the Chinese version of the Perceived Stress Scale (CPSS), revised by Yang and Huang (2003). This scale consists of 14 items reflecting feelings of tension (e.g., “I felt nervous and stressed”) and loss of control due to stress (e.g., “Felt that I could not control the important things in my life”), with responses rated from 1 (completely disagree) to 5 (completely agree). The total score is calculated by summing the scores of all items, with higher scores indicating higher perceived stress. Cronbach’s α in this study was 0.734.

#### 2.3.2. Adolescent Social Anxiety Scale

Social anxiety was assessed using the abbreviated version of the SAS-A (Social Anxiety among Adolescents), introduced by Sun et al. (2017). This scale contains 12 items, with responses ranging from 1 (completely disagree) to 5 (completely agree), covering three dimensions: fear of negative evaluation (e.g., “I worry about what others think of me”), social avoidance and distress in unfamiliar situations (e.g., “I feel nervous when talking to peers I don’t know well”), and social avoidance and distress in general situations (e.g., “I feel shy even when I am with people I know very well”). It is an effective tool for investigating and screening the social anxiety of Chinese junior high school students (Sun et al., 2017). Cronbach’s α was 0.916.

#### 2.3.3. Avoidance and Fusion Questionnaire for Youth

The Avoidance and Fusion Questionnaire for Youth (AFQ-Y8) was developed by Greco et al. (2008). Y. H. Chen et al. (2019) translated the scale into Chinese and initially tested its reliability and validity in this population. This scale includes 8 items that focus on two dimensions: experiential avoidance (e.g., “When I feel bad, I stop doing things that are important to me”) and cognitive fusion (e.g., “The bad things I think about myself must be true”). Responses are rated on a 5-point scale from 1 (completely disagree) to 5 (completely agree). Higher scores reflect greater psychological inflexibility. Cronbach’s α in this study was 0.856.

#### 2.3.4. Adolescent Questionnaire for Assessing Problematic Social Media Use

The questionnaire developed by Jiang (2018), comprising 20 items and employing a five-point scoring scale (1 = completely disagree, 5 = completely agree), was used to assess PSMU among junior high school students. It evaluates five dimensions: increased engagement stickiness (e.g., “Unconsciously check mobile apps and browse social feeds many times a day”), physical harm (e.g., “Frequent and prolonged use of social media often causes dry eyes and visual fatigue”), missed opportunity anxiety (e.g., “I worry about missing out on information if I don’t check WeChat or Weibo for a while”), impaired cognitive function (e.g., “The time I spend on deep thinking has decreased compared to before”), and guilt (e.g., “I often regret wasting time on my phone because it delays my important task”). This instrument is well-adapted to the realities of Chinese adolescents, demonstrating strong reliability and validity with a Cronbach’s alpha coefficient of 0.925 in this study, making it an effective tool for measuring PSMU in junior high school students.

### 2.4. Statistical Analysis

The study utilized SPSS 27.0 and PROCESS macro Model 6 to conduct descriptive statistical analysis, correlation analysis, and mediation effect testing. A bias-corrected bootstrap method with 5000 resamples was employed to estimate the 95% confidence intervals for the indirect effects, thereby examining the mediating roles of psychological inflexibility and PSMU in the relationship between perceived stress and social anxiety (Hayes & Scharkow, 2013).

Common method bias was examined using Harman’s single-factor method. The results showed that the first factor accounted for 28.3% of the cumulative variance, which is below the critical threshold of 40% (Tang & Wen, 2020), indicating that no significant common method bias was present. In addition, procedural remedies such as reverse-scored items were also adopted.

## 3. Results

### 3.1. Preliminary Analysis

Table 1 presents the descriptive statistics and correlation analysis of the variables. Perceived stress was significantly positively correlated with social anxiety, psychological inflexibility, and PSMU. Gender was positively correlated with perceived stress, social anxiety, and psychological inflexibility, but showed no significant association with PSMU. Age was positively correlated with all variables except gender.

### 3.2. Chain Mediation Analysis

**Figure 2:**
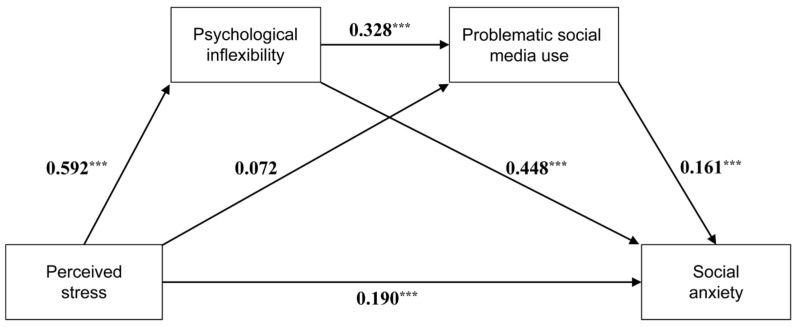
The Chain Mediation Model of Psychological Inflexibility and Problematic Social Media Use. Note. *** *p* < 0.001.

After controlling for gender and age, the study further analyzed the association between perceived stress and social anxiety, with psychological inflexibility and PSMU as mediating variables. As shown in Figure 2, the direct path from perceived stress to social anxiety was significant (*β* = 0.190, *p* < 0.001), indicating that perceived stress significantly and positively predicts social anxiety. Perceived stress significantly and positively predicted psychological inflexibility (*β* = 0.592, *p* < 0.001), and psychological inflexibility significantly and positively predicted social anxiety (*β* = 0.448, *p* < 0.001), suggesting that psychological inflexibility mediates the relationship between perceived stress and social anxiety. PSMU significantly and positively predicted social anxiety (*β* = 0.161, *p* < 0.001), indicating that PSMU also mediates the relationship between perceived stress and social anxiety. Moreover, psychological inflexibility significantly and positively predicted PSMU (*β* = 0.328, *p* < 0.001), demonstrating a chain mediating role of psychological inflexibility and PSMU between perceived stress and social anxiety.

Consistent with these findings, the bootstrap mediation analysis (see Table 1) showed that the indirect effect of perceived stress → psychological inflexibility → social anxiety was 0.422, with a 95% confidence interval that did not include 0, indicating a significant mediating effect of psychological inflexibility. The indirect effect of perceived stress → PSMU → social anxiety was 0.019, with a 95% confidence interval that included 0, indicating that the mediating effect of PSMU was not significant. The indirect effect of perceived stress → psychological inflexibility → problematic social media use → social anxiety was 0.050, indicating a significant chain mediating effect of psychological inflexibility and PSMU between perceived stress and social anxiety, Table 2.

Accordingly, H1, H2, and H4 can be assumed to hold, while H3 is not supported.

## 4. Discussion

This study explored the effect of perceived stress on Chinese junior high school students’ social anxiety through psychological inflexibility and PSMU. The results showed that perceived stress was significantly associated with social anxiety in junior high school students. Furthermore, psychological inflexibility and PSMU exhibited a chain mediating effect between perceived stress and social anxiety.

### 4.1. Relationship Between Perceived Stress and Social Anxiety

Consistent with previous research (Ni et al., 2016; van Loon et al., 2022), junior high school students who reported higher levels of perceived stress also experienced greater tension, unease, and fear in social situations.

According to the conservation of resources theory (Halbesleben et al., 2014), persistent resource depletion weakens an individual’s ability to cope with challenges. Under the multiple pressures of academic and daily life, junior high school students experience substantial depletion of their cognitive and psychological resources, making them more inclined to avoid situations requiring social interaction. This maladaptive coping style not only induces more negative self-evaluations but also exacerbates social anxiety (Zhu et al., 2024). Furthermore, perceived stress leads to physiological changes such as increased heart rate and sweating, which are easily misinterpreted by junior high school students as signals of anxiety, thereby further reinforcing their experience of anxiety (Liu et al., 2023).

### 4.2. The Independent Mediating Effects of Psychological Inflexibility and Problematic Social Media Use

This study indicated that perceived stress can exacerbate individual social anxiety by increasing the level of psychological inflexibility among junior high school students. The research found that students with higher levels of psychological inflexibility generally exhibit more severe social anxiety problems (G. Zhang, 2021). Such students tend to adopt automated, rigid, and stereotyped cognitive schemas to interpret stressful life events, displaying strong stubbornness in social situations and difficulty accepting differing opinions from others. When faced with stress, they may become fused with negative self-judgments and avoid social situations to prevent potential loss of face. This rigid avoidance prevents them from learning that social interactions can be safe and rewarding, thereby perpetuating and intensifying social anxiety.

The study found that PSMU did not independently mediate the relationship between perceived stress and social anxiety. Thus, Hypothesis 3 was not supported. This null finding is noteworthy and suggests that, among junior high school students, simply using social media excessively or frequently does not necessarily translate into increased social anxiety. Rather, the effects of social media use on anxiety may depend on why and how adolescents use these platforms (Li et al., 2018).

Junior high school students face intense academic pressure and parental expectations. Their social media use may often serve as a transient, recreational escape or a way to follow peer trends, rather than a genuine substitute for real-world social interaction or a means of relationship maintenance. The compensatory internet use model (Kardefelt-Winther, 2014) posits that negative outcomes depend on the underlying motivations for use. Indeed, some studies have found that only certain types of use (e.g., social comparison, fear of missing out) are associated with increased anxiety (Li, 2024).

### 4.3. The Chain-Mediated Effect of Psychological Inflexibility and Problematic Social Media Use

This study confirmed Hypothesis 4. Although the independent mediating effect of PSMU did not reach significance, Psychological inflexibility plays a key role in problematic social media use. Specifically, individuals with higher levels of psychological inflexibility often struggle to adapt flexibly to changing circumstances (Y. Chen et al., 2019). When junior high school students perceive stress and fall into a state of psychological inflexibility, their ability to respond flexibly to external situations significantly declines. They tend to avoid experiences or situations that may trigger negative feelings and instead turn to social media to seek positive experiences. According to Davis’s (2001) cognitive-behavioral model of pathological internet use, individuals with maladaptive cognitions are more likely to develop problematic use behaviors. For junior high school students with weaker psychological adaptability, social media provides a seemingly safe and controllable environment, thus becoming a tool for escaping real-world challenges and negative emotions. For example, a student who feels overwhelmed by exam pressure and holds rigid beliefs about failure may escape to short-video platforms, avoiding face-to-face interactions. Over time, this PSMU, driven by experiential avoidance, not only impairs real-life social skills but also increases sensitivity to social evaluation, thereby exacerbating social anxiety and ultimately forming a vicious cycle. Therefore, improving junior high school students’ rigid psychological coping patterns is particularly crucial for reducing their level of social anxiety.

### 4.4. Intervention Recommendations

The findings of this study provide empirically grounded guidance for interventions aimed at reducing social anxiety among junior high school students. Given the direct positive effect of perceived stress on social anxiety, mitigating social anxiety should begin with stress management. In particular, early intervention targeting stress appraisal and coping skills may help reduce this direct pathway. Schools may implement universal stress reduction programs that help students reappraise stressful events and develop adaptive coping strategies, such as cognitive restructuring based on the cognitive appraisal theory and relaxation techniques, including mindfulness breathing and progressive muscle relaxation.

In addition to perceived stress, psychological inflexibility is also an important variable that requires intervention. Acceptance and Commitment Therapy (ACT) has demonstrated efficacy in reducing psychological inflexibility and alleviating social anxiety (Gloster et al., 2020; Niles et al., 2014). ACT group counseling can employ training methods tailored to the physical and psychological developmental stages of junior high school students, such as cognitive defusion, acceptance of discomforting emotions, and value clarification, to improve their cognition and behavior, thereby enhancing psychological flexibility.

Moreover, concerning problematic social media use, our findings suggest that simply reducing screen time or frequency may be insufficient. Thus, researchers and practitioners should focus on the psychological motivations and cognitive patterns underlying social media behavior rather than merely on screen time or frequency.

To facilitate the implementation of interventions, multi-stakeholder collaboration is of utmost importance. Mental health teachers and school administrators should receive systematic ACT training to develop short-term group intervention programs tailored for junior high school students. Parents need to recognize that psychological inflexibility is a key risk factor for social anxiety and should foster an inclusive and open family communication environment rather than simply banning electronic devices. Educational authorities, for their part, may consider integrating psychological flexibility training into the mental health curriculum, thereby building a comprehensive prevention network that extends from the individual level to the broader social environment.

### 4.5. Research Significance and Limitations

This study elucidates the intrinsic mechanism by which perceived stress influences social anxiety in junior high school students through a chain-mediated effect involving psychological inflexibility and PSMU, providing theoretical perspectives and empirical evidence for intervention strategies.

This study has several limitations. First, the cross-sectional design cannot establish causal relationships or temporal sequences among the variables. Second, the sample has limited representativeness, making it difficult to fully capture the circumstances of junior high school students from different regions and family educational backgrounds. Third, the use of self-report questionnaires may introduce subjective bias. Fourth, the study did not distinguish between active and passive social media use, which may obscure their differential associations with social anxiety. Fifth, our model focused exclusively on risk factors (psychological inflexibility and PSMU) and did not include protective variables (e.g., resilience, social support) that could buffer the effects of perceived stress.

Future research should address these limitations. Longitudinal designs are needed to verify the directionality of the proposed pathways, compare differences across regions and developmental stages, and examine the moderating role of chain mediation. Researchers should also differentiate between active and passive social media use and further explore other potential mediating or moderating variables in the relationship between perceived stress and social anxiety. Future research should also incorporate positive psychology constructs (e.g., self-compassion, dispositional mindfulness, resilience) to examine how they may buffer the transition from perceived stress to psychological inflexibility and social anxiety. Methodologically, multi-informant data (e.g., parent reports, teacher evaluations, peer assessments) combined with experimental and in-depth interview methods would help test causal pathways, elucidate underlying mechanisms, and minimize the influence of confounding factors.

## 5. Conclusions

This study examined the relationship between perceived stress and social anxiety among junior high school students. The results show that perceived stress directly predicts social anxiety and may be indirectly related to it through the chain mediation of psychological inflexibility and PSMU. Specifically, perceived stress may trigger psychological inflexibility, which in turn may promote PSMU and ultimately may exacerbate social anxiety. These findings highlight that stress management, psychological flexibility training, and the cultivation of healthy social media use behaviors can effectively reduce social anxiety in junior high school students. Overall, this study provides empirical evidence for school mental health educators to optimize intervention strategies, aiming to help adolescents alleviate psychological stress, reduce social anxiety, and promote social adaptation and overall well-being.

## Figures and Tables

**Table 1:** Descriptive Statistics and Correlation Analysis for Each Variable (*n* = 682).

Variables	*M* ± *SD*	1	2	3	4	5	6
1. Gender	–	1					
2. Age	14.46 ± 0.50	0.001	1				
3. Perceived Stress	2.96 ± 0.54	0.079 *	0.108 **	1			
4. Social Anxiety	2.31 ± 0.89	0.142 ***	0.120 **	0.502 ***	1		
5. Psychological Inflexibility	2.45 ± 0.84	0.156 ***	0.091 *	0.581 ***	0.630 ***	1	
6. Problematic Social Media Use	1.90 ± 0.68	0.041	0.179 ***	0.276 ***	0.391 ***	0.380 ***	1

Note. Gender was dummy coded such that (0 = female, 1 = male). * *p* < 0.05. ** *p* < 0.01.*** *p* < 0.001.

**Table 2:** Direct and Indirect Association Measuring Paths from Perceived Stress to Social Anxiety.

Effect Type	Effect Size	BootSE	Boot 95% CI	Ratio of Total
Direct Effect	0.315	0.058	[0.200, 0.429]	39.08%
Perceived Stress → Psychological Inflexibility → Social Anxiety	0.422	0.050	[0.325, 0.522]	52.36%
Perceived Stress → Problematic Social Media Use → Social Anxiety	0.019	0.013	[−0.045, 0.049]	2.36%
Perceived Stress → Psychological Inflexibility → Problematic Social Media Use → Social Anxiety	0.050	0.015	[0.024, 0.081]	6.20%
Total Indirect effect	0.491	0.046	[0.403, 0.586]	60.92%
Total Effect	0.806	0.055	[0.698, 0.914]	100.00%

## Data Availability

The dataset generated and analyzed for the current study is not publicly available but is available from the corresponding author on reasonable request.
